# Polychlorinated Biphenyls (PCBs): Risk Factors for Autism Spectrum Disorder?

**DOI:** 10.3390/toxics8030070

**Published:** 2020-09-17

**Authors:** Harmanpreet Kaur Panesar, Conner L. Kennedy, Kimberly P. Keil Stietz, Pamela J. Lein

**Affiliations:** 1 Department of Molecular Biosciences, School of Veterinary Medicine, University of California, Davis, CA 95616, USA; hkpanesar@ucdavis.edu; 2 Department of Comparative Biosciences, School of Veterinary Medicine, University of Wisconsin, Madison, WI 53706, USA; clkennedy3@wisc.edu

**Keywords:** axons, bladder dysfunction, dendritic arborization, dendritic spines, calcium signaling, neuronal connectivity, persistent organic pollutants, ryanodine receptor, synapses

## Abstract

Autism spectrum disorder (ASD) includes a group of multifactorial neurodevelopmental disorders defined clinically by core deficits in social reciprocity and communication, restrictive interests and repetitive behaviors. ASD affects one in 54 children in the United States, one in 89 children in Europe, and one in 277 children in Asia, with an estimated worldwide prevalence of 1–2%. While there is increasing consensus that ASD results from complex gene x environment interactions, the identity of specific environmental risk factors and the mechanisms by which environmental and genetic factors interact to determine individual risk remain critical gaps in our understanding of ASD etiology. Polychlorinated biphenyls (PCBs) are ubiquitous environmental contaminants that have been linked to altered neurodevelopment in humans. Preclinical studies demonstrate that PCBs modulate signaling pathways implicated in ASD and phenocopy the effects of ASD risk genes on critical morphometric determinants of neuronal connectivity, such as dendritic arborization. Here, we review human and experimental evidence identifying PCBs as potential risk factors for ASD and discuss the potential for PCBs to influence not only core symptoms of ASD, but also comorbidities commonly associated with ASD, via effects on the central and peripheral nervous systems, and/or peripheral target tissues, using bladder dysfunction as an example. We also discuss critical data gaps in the literature implicating PCBs as ASD risk factors. Unlike genetic factors, which are currently irreversible, environmental factors are modifiable risks. Therefore, data confirming PCBs as risk factors for ASD may suggest rational approaches for the primary prevention of ASD in genetically susceptible individuals.

## 1. Introduction

Autism spectrum disorder (ASD) includes a group of multifactorial neurodevelopmental disorders defined clinically by core deficits in social reciprocity and communication, restrictive interests and repetitive behaviors. The severity of core symptoms, the expression of co-morbidities, which include intellectual disability, seizures, anxiety, gastrointestinal symptoms, and immunological abnormalities, and the response to treatment vary considerably between individuals diagnosed with ASD [1,2]. The Center for Disease Control (CDC) estimates the current prevalence of ASD among 8-year-old children in the United States to be 1 in 54 (https://www.autism-society.org). A project funded by the European Parliament and managed by the European Commission reported in December 2018 that the prevalence of ASD in Europe ranged from 4.4 to 19.7 per 1000 children aged 7–9 years old, with an averaged estimated prevalence of 12.2 per 1000 (or 1 in 89 children) (http://asdeu.eu/wp-content/uploads). A recent meta-analysis of literature published up to August 6, 2018, concluded that the pooled estimated of ASD prevalence in Asia was 3.6% (95% CI: 0.16–0.79%) [3]. Large-scale surveys estimate the median worldwide prevalence of ASD to be 1–2% [4,5]. When considered in the context of the tremendous costs of ASD to the affected individual, their families, and society [6,7,8], these statistics underscore the need to identify factors that confer ASD risk and/or modify symptom severity.

Until recently, research on the etiology of ASD largely focused on genetic causes. Genetic research has identified a strong hereditary component for ASD [9,10,11] and shown that the candidate genes most strongly associated with ASD encode proteins that regulate the patterning of neuronal networks during development [12,13,14,15]. However, genetic research has also shown that genes linked to ASD rarely segregate in a simple Mendelian fashion [10] and that single genetic anomalies account for a very small proportion of cases [14,16]. Moreover, there is incomplete concordance of ASD diagnosis in monozygotic twins [16], and even in genetic syndromes strongly associated with ASD, a significant percentage of carriers do not express autistic phenotypes [16,17]. These observations are consistent with a model in which environmental factors act as modifiers of ASD risk genes.

The rapid rise in ASD prevalence over the past several decades [14,18] also provides compelling evidence in support of the hypothesis that environmental factors interact with genetic susceptibilities to determine ASD risk. While improved detection and broadening of diagnostic criteria for ASD contribute to the progressive rise in ASD prevalence, several studies have concluded that factors other than diagnostic drift account for more than half of new cases, indicating a true increase in the number of individuals diagnosed with ASD [19,20,21]. Findings from large twin studies suggest that environmental factors account for approximately 50–60% of ASD cases [22,23]. A critical influence of environmental factors on ASD risk and severity provides a plausible explanation for not only the dramatic increase in ASD prevalence, but also the significant clinical heterogeneity that is a hallmark characteristic of this disorder.

In contrast to genetic risks, which are currently irreversible, environmental factors are modifiable risk factors. Identifying specific environmental factors that influence risk for ASD may suggest rational approaches for the primary prevention of the symptoms associated with the disorder. Progress has been made in identifying environmental risk factors for ASD, including advanced paternal age at conception, complications during pregnancy, maternal diet, and prenatal exposure to psychotropic drugs [24,25]. While environmental chemicals are widely posited to also contribute to ASD risk [14,26,27,28], it has been difficult to demonstrate this in human studies. This reflects two major challenges in the field. First, ASD is phenotypically heterogeneous with children diagnosed as having ASD based on observable symptom clustering, not causal pathways [29,30,31]. Second, the complexity of heritable risk factors contributing to ASD likely creates a range of sensitivities to environmental risk factors [32], which masks clear associations between exposure and diagnosis. To overcome these challenges and to inform more targeted epidemiologic studies, experimental models are being leveraged to identify environmental chemicals that modulate the same signaling pathways and neurodevelopmental events as ASD susceptibility genes [32,33]. Such studies have identified polychlorinated biphenyls (PCBs) as putative ASD risk factors [28].

PCBs are a class of 209 structurally related chemicals, known as congeners, comprised of a biphenyl with variable numbers of chlorine substitutions in varying positions on the benzene rings. Congeners with ≤4 chlorine substituents are referred to as lower-chlorinated PCBs; congeners with >4 chlorine substituents, as higher-chlorinated PCBs [34]. Lower-chlorinated PCBs tend to be more volatile while higher-chlorinated PCBs tend to bioaccumulate [34]. PCBs can also be classified according to their planar structure, which is determined by the position of the chlorine substituents on the biphenyl. In the absence of any chlorines in the *ortho* position, the rings of the biphenyl backbone assume a coplanar geometry, whereas, in the presence of one to four chlorines in the *ortho* position, the rings assume an increasingly noncoplanar geometry. Coplanar congeners can bind the aryl hydrocarbon receptor (AhR), and, since the AhR is the canonical receptor for 2,3,7,8,-tetrachlorodibenzo-p-dioxin (TCDD), coplanar PCB congeners are often referred to as dioxin-like (DL) PCBs [35]. In contrast, noncoplanar congeners have little to no binding affinity for the AhR and thus are referred to as non-dioxin-like (NDL) PCBs.

Beginning in the late 1920s, PCBs were synthesized in large amounts predominantly for use as coolants, lubricants, and stabilizers in diverse industrial and commercial applications. However, in response to increasing evidence of their environmental persistence and concerns regarding the carcinogenic potential of the DL PCBs, the United States banned further PCB production in 1979. In 2001, the signatory countries of the Stockholm Convention on Persistent Organic Pollutants (POPs) instituted a more global ban on PCB production, which was appended in 2008 and 2014. Despite the cessation of large-scale PCB production, humans continue to be exposed to legacy PCBs (a term used to refer to the congeners present in commercial mixtures) because these compounds continue to be released from PCB-containing transformers and capacitors still in use, hazardous waste sites, and construction materials used in buildings erected prior to the ban on PCB production [36]. In addition, data emerging over the past decade demonstrate widespread human exposure to not only legacy but also “contemporary” PCB congeners that were not present in commercial PCB mixtures but are unintentional byproducts of current manufacturing processes for pigments [34,36,37]. These non-legacy PCBs are detected in indoor and outdoor environments and in human tissues, and the most likely source is off gassing from common household paints, varnishes, and caulking [36,37,38].

It is thought that humans are predominantly exposed to the higher-chlorinated PCBs via the ingestion of contaminated foods, and to lower-chlorinated PCB via inhalation [34]. However, recent reports demonstrating the prevalence of the lower-chlorinated congener PCB 11 in commercial milk products in northern California [39], and evidence that PCB 95, a higher-chlorinated congener, is the second most abundant PCB detected in the air in schools in the United States [40], suggest that humans may be exposed to both higher- and lower-chlorinated PCBs via diet and inhalation [41]. PCBs are detected in human tissues globally with the highest PCB body burdens detected in the Inuit population in Greenland whose adipose tissue contains 3–34-fold higher amounts of PCBs than comparable samples from individuals living in Quebec City, Canada [42]. Total PCB levels in human postmortem samples (adipose, brain and liver) from Greenland were approximately 66 ng/g wet weight (ww) [42]. In contrast, mean PCB concentrations ranging from 10.6 to 35.3 ng/g have been reported in liver, muscle, kidney, and brain samples from men and women in Belgium [43], while median levels of 1.5 ng/g ww (range <LOD—18.5 ng/g ww; based on eight congeners) were detected in postmortem human tissue samples from the United States [44].

PCBs readily cross the placenta and are transferred through breast milk [45]. Thus, PCBs are a particular concern for the developing brain, which develops rapidly during gestation and early life. National Health and Nutrition Examination Survey (NHANES) data confirm widespread exposure to PCBs among women of childbearing age currently living in the United States [46], including the lower-chlorinated contemporary PCBs [36,37,47,48,49]. The sum of 34 total PCB congeners in the blood of pregnant women living in northern California was determined to be 16.1–148 ng/g lipid [50], while the sum of 17 PCB congeners was 33.63–662.34 ng/g lipid [51]. Similar results have been reported in countries around the world. Analysis of PCB levels in blood from pregnant women in two different Japanese cohorts reported the sum total PCBs (13 examined) of 17.8–362 ng/g lipid [52], and 20–210 ng/g lipid (29 congeners examined [53]. Median levels of PCB 153, widely used as an indicator PCB, in the serum of pregnant mothers in Greenland were 107 ng/g lipid [42], while, in the Ukraine, they were 27 ng/g lipid; the latter was associated with cognitive defects in the children [54]. Non-legacy PCBs are detected in the plasma of pregnant women at levels ranging from 0.005 to 1.717 ng/mL [37,49]. PCBs have also been detected in human cord blood (56.29 ng/g lipid [55]) and in brain tissue from human fetuses and young children (22–122 ng/g lipid [56] and 3.72–67.15 ng/g lipid [44]). Interestingly, levels of PCB 95 in post-mortem brain tissue from children with ASD were found to be significantly higher than levels in brain tissue from neurotypical children [44].

Below, we summarize what is currently known about the effects of developmental PCB exposure on the developing human brain and discuss mechanisms of PCB developmental neurotoxicity that map onto signaling pathways and neurodevelopmental outcomes associated with ASD. We also propose mechanisms by which PCBs may interact with ASD risk genes to increase the risk and/or severity of ASD and discuss the potential for PCBs to contribute to not only the core symptoms, but also common comorbidities of ASD using bladder dysfunction as an example.

## 2. Neurobehavioral Effects of Developmental PCB Exposures

PCBs first gained attention as human developmental neurotoxicants as a result of the accidental ingestion of cooking oil contaminated with PCBs in Yusho, Japan, in 1968 [57] and in Yu-Cheng, Taiwan, in 1979 [58]. Children born to women who ingested PCB-contaminated cooking oil while pregnant had a significantly increased incidence and severity of cognitive and psychomotor deficits. While these incidents involved high-level PCB exposures, subsequent epidemiologic studies of infants and children exposed to lower levels of PCBs also found an association between PCBs and neuropsychological deficits (reviewed in [59,60,61,62]). The conclusions of these earlier reviews were recently extended by a summary of the literature on human PCB developmental neurotoxicity published in English-language journals between 1990 and 2018 [45]. The authors of this summary identified 29 papers that met the following inclusion criteria: (1) sample size of ≥100 subjects; (2) prospective cohort study design that measured prenatal PCB exposures; (3) quantification of PCBs in biospecimens, primarily maternal serum or plasma collected during pregnancy or at delivery, including from the umbilical cord and placenta; and (4) subjects were ≥3 years of age “as neurodevelopmental outcomes can be measured more reliably in older children”. These 29 studies described populations from diverse geographic regions, including North America (the United States, Canada, and Greenland), Asia (Japan), and Europe (Ukraine, Spain, the Netherlands, Germany, the Faroe Islands, Denmark, and Belgium). The studies were also diverse with respect to cohort birth year, reflecting differing PCB congener profiles during pregnancy as levels of legacy PCBs have slowly decreased since the ban on PCB production, while environmental levels of contemporary PCBs have increased [63]. The studies included in the summary also varied widely with respect to the number and type of PCB congeners measured in biospecimens, as well as the limit of detection of the methods used to quantify PCBs [45].

The review identified 12 publications from 9 different cohorts that examined impacts of PCBs on cognitive function among children aged 3–11 years [45]. Most (8 of 12) found associations of developmental PCB exposure with deficits in at least one measure of cognition. Two studies that identified an association of PCBs with decreased IQ at 3 years of age found this association weakened by the time children were 4 and 6 years of age. A total of 17 studies from 11 different cohorts examined associations of prenatal PCBs with attention, behavioral regulation and social behavior among 3–12-year-old children [45]. The majority (10 of 17) reported PCB-related associations with impulse control, hyperactivity, and attention. Only two of 17 studies examined PCBs in relation to social behavior and autistic traits, with one study reporting that total PCB levels were associated with fewer autistic traits [64], and the other reporting congener-specific associations with autistic traits [65].

Overall, this focused summary concluded that most studies found prenatal PCB exposures were related to poorer cognitive function and behavior problems [45]. As acknowledged by the authors, a limitation of their review was that associations with specific PCB congeners or mechanism-based classes of congeners were not evaluated, largely because most epidemiologic studies report total sum PCB levels or levels of “indicator” PCBs. The significance of this limitation is underscored by a recent study suggesting that NDL PCBs with activity at the ryanodine receptor (RyR), but not DL PCBs or total PCBs, are marginally positively associated with ASD [51]. The genetic substrate likely also influences the impact of PCBs on ASD-relevant outcomes, as illustrated by a recent pilot study not included in the focused summary that identified a trend towards a positive association between PCB 153 and ASD in individuals with a deletion mutation in the gene encoding glutathione transferase but not in individuals who did not have this mutation [66]. Also not included in the summary, because the age of the child at the time of ASD diagnosis was not specified, was a 2017 study that found ASD risk was elevated for a number of PCB congeners, particularly for the highest vs. lowest quartile of PCB138/158 (AOR = 1.79; 95% CI: 1.10, 2.71) and PCB153 (AOR = 1.82; 95% CI: 1.10, 3.02), and for the highest deciles of other congeners examined in secondary analyses [67]. Finally, a 2020 study reported an association between plasma PCB concentrations measured during pregnancy and increased incidence of autistic behaviors in children aged 3–4 years old when the data were analyzed using Bayesian predictive odds ratios [68]. When considering these additional studies that have examined the impact of prenatal PCB on ASD phenotypes together with the with studies included in the review, most (five of six) report that PCBs increase the expression of autistic traits.

Preclinical studies in non-human primate and rodent studies confirm that developmental PCB exposures, primarily via the maternal diet or direct oral exposure in young animals, negatively impact cognitive function and occur in the absence of adverse effects on reproduction or birth outcomes (reviewed in [69,70,71,72]). While many of the earlier studies focused on the developmental neurotoxicity of legacy commercial mixtures, more recently, the neurodevelopmental impacts of individual PCB congeners or custom mixes based on PCB congener profiles documented in human tissues have been investigated. These studies are summarized in a recent review of animal studies published in the past decade that employed exposure paradigms relevant to humans, in terms of both the dose and exposure route [73]. The authors of the review concluded that the developmental neurotoxicity associated with exposures to legacy PCB mixtures is primarily mediated by NDL congeners. The relevance of this conclusion is suggested by reports indicating that NDL PCBs represent a significantly greater percentage of the PCBs detected in contemporary samples of human serum, adipose tissue, breast milk, as well as brain tissue from children with ASD [44,51,74].

While cognitive function has been the major focus of animal studies investigating PCB effects on behavior, there are emerging reports of PCB effects on behavioral domains of more direct relevance to the core symptoms of ASD, such as social interactions and communication. While rodent models cannot recapitulate many aspects of human social interaction [75], they can capture core aspects of sociability [76]. Sociability in rodents, defined as an animal’s preference for investigating and spending time with a conspecific animal, can be directly tested in a three-chamber social approach test and similar tasks [75,77,78,79]. Ultrasonic vocalizations (USVs) and sociosexual choice are also frequently used to assess sociability and communication in rodent models [80,81]. The animal literature describing the effects of developmental PCB exposure on social behavior was recently reviewed [69,73]. Briefly, five studies, one in mice [82] and four in rats [83,84,85,86], have been published that describe PCB effects on various metrics of sociability. In the mouse study, animals were exposed via the maternal diet to a mixture of six NDL PCB congeners found in human blood at levels approximating those in the human diet (10 or 1000 ng/kg/d). This exposure reduced nose-to-nose interactions between males, but enhanced sociability and social approach in females and males [82]. In contrast, the four rats studies (three of which were performed in the same laboratory), demonstrated that perinatal exposures to either a mixture of PCBs 47 and 77 [84] or Aroclor 1221 [83,85,86] generally decreased sociability metrics in males and had mixed effects in females depending on the dose and timing of administration [73]. These observations are consistent with the sex bias of ASD; however, it is not possible to determine from these studies whether the effects are attributable to a specific mechanistic or structural subgroup of PCBs. Overall, there is a need for more comprehensive structure–activity relationship (SAR) studies of the neurobehavioral effects of developmental PCB exposures to determine whether the profile of developmental neurotoxicity varies in a congener- and sex-specific manner across different behavioral domains.

A key unanswered question in the field is the role of xenobiotic metabolism in determining neurotoxic outcomes following developmental PCB exposures. The cytochrome p450 enzymes play a major role in PCB metabolism, converting the parent compounds to hydroxylated PCBs, which in turn can be further metabolized to glucuronide, sulfate and other conjugates [34,87,88]. PCB metabolites are detected in serum from occupationally exposed humans [89] and pregnant women [90]. Hydroxylated PCB exposure during pregnancy has been associated with impaired motor development and cognitive function in children [91,92,93]. In adolescent children, exposure to hydroxylated PCBs during gestation was negatively associated with long-term memory while other parameters, such as auditory attention, had less frequent sub-optimal scores with higher exposures to hydroxylated PCBs [90]. Another recent study observed a positive association between hydroxylated PCBs and mental development in children 30 months of age, while hydroxylated PCB 187 was associated with a lower mental development index at 18 months of age [94]. The reasons for the discrepant findings of the association between hydroxylated PCB exposure and cognitive performance are not known but could be linked to the diverse effects of PCB metabolites on dendritic architecture (see Section 3) and/or endocrine disruption. Hydroxylated PCBs have a differential ability to agonize or antagonize steroid hormone pathways [95], and in vitro evidence indicates that estrogenic effects of hydroxylated PCBs can be greater than that of their parent compound [96]. Maternal serum levels of hydroxylated PCBs have also been associated with free thyroxin levels in neonates [97], suggesting disruption of thyroid hormone signaling may influence the effects of hydroxylated PCB on cognition. Further complicating the interpretation of the impact of metabolism on PCB developmental neurotoxicity, studies in mice have shown that not only can the tissue distribution of parent PCBs and their metabolites differ, but for chiral PCBs, which includes many RyR-active PCBs, the enantiomeric enrichment of parent versus metabolites can differ in a tissue-specific manner [98]. Furthermore, PCB metabolism can be altered by pregnancy [99]. These studies highlight the need for a better understanding of how the biotransformation of PCBs contributes to overall NDD risk.

## 3. Mechanisms of PCB Developmental Neurotoxicity Relevant to ASD

Recent advances in defining the molecular and cellular pathology of ASD point to altered patterns of neuronal connectivity in the developing brain as the neurobiological basis for the clinical symptoms associated with this disorder [13,28,100,101,102]. Axonal and dendritic morphology are critical determinants of neuronal connectivity [103]. The number, length and branching patterns of these processes determine the pattern of synaptic connections, which, in turn, regulates the distribution of information within the nervous system. Experimental evidence indicates that even subtle perturbations of temporal or spatial aspects of axonal and dendritic growth can cause persistent changes in synaptic patterning in the developing brain and adversely impact neurobehavior [104,105,106,107]. Clinical evidence indicates that both axonal and dendritic growth are altered in relevant brain regions of autistic individuals [101,108,109,110,111,112].

PCBs have been reported to alter in vivo axonal outgrowth in a rat model of developmental PCB exposure [113]. Rats were exposed throughout gestation and lactation to Aroclor 1254 in the maternal diet at 125 ppm and then maintained on chow containing 125 ppm Aroclor 1254 after weaning until being euthanized. Timm’s silver sulfide staining revealed that Aroclor 1254 significantly reduced the length of II-P mossy fibers in 16-, 30- and 60-day-old rats. In contrast, Aroclor 1254 had no effect on hilar or suprapyramidal mossy fibers or on cortical thickness. The reason(s) for the selective sensitivity of granule cells that extend II-P mossy fibers has yet to be determined.

The effects of developmental exposures to Aroclor 1254 on dendritic morphogenesis have also been evaluated in vivo [114,115,116]. In three independent cohorts of Long–Evans rats, pups were exposed to Aroclor 1254 at 6 mg/kg/d in the maternal diet throughout gestation and lactation. In one study [116], a lower dose of Aroclor 1254 (1 mg/kg/d) was also tested. In two of the three studies, Golgi staining results indicated that Aroclor 1254 significantly altered dendritic morphology in the brains of offspring [114,116]. In one of these two studies [114], Aroclor 1254 at 6 mg/kg/d caused a robust age-related increase in the rate of dendritic growth in Purkinje cells of the cerebellum and pyramidal neurons of the CA1 hippocampus. Specifically, at postnatal day (PND) 22, dendrites were significantly less complex in PCB-exposed animals, but by PND 60, dendritic growth was equal to or significantly greater than that observed in vehicle controls [114]. In the second study [116], basal dendritic arborization in cerebellar Purkinje cells and neocortical pyramidal neurons of PND 31 male offspring was significantly increased by Aroclor 1254 exposure; however, experience-dependent dendritic growth was significantly stunted in these groups. These dendritic effects were observed in the cerebellum of the 1 mg/kg/d, but not 6 mg/kg/d, exposure group. In contrast, dendritic arborization in the neocortex was significantly enhanced in both the 1 and 6 mg/kg/d exposure groups relative to vehicle controls, but the effect was significantly greater in the 1 mg/kg/d group. Interestingly, this study also found that exposure to Aroclor 1254 at 1 mg/kg/d, but not 6 mg/kg/d, caused significant spatial learning and memory deficits [116]. The third study [115], which only examined dendritic arborization in cerebellar Purkinje cells at PND 21, found no effects of 6 mg/kg/d Aroclor 1254 on dendritic morphology. While this finding is consistent with the second study [116], it is at odds with the first study [114], which reported that developmental exposure to 6 mg/kg/d Aroclor 1254 altered the dendritic morphology of Purkinje cells. Collectively, these studies indicate that developmental PCB exposure appears to modulate the rate of dendritic growth in the postnatal brain. Thus, the discrepant findings between studies may reflect differences in the ages at which dendritic morphology was evaluated.

Developmental exposure to PCB 95, a legacy NDL congener, was similarly shown to enhance dendritic arborization in vivo [117]. Rats were exposed throughout gestation and lactation to 0.1, 1, or 6 mg/kg PCB 95 in the maternal diet. At PND 38, brains were Golgi-stained to quantify the dendritic arbors of hippocampal CA1 pyramidal neurons. Similar to Aroclor 1254, PCB 95 enhanced dendritic arborization in a non-monotonic dose-related manner with significantly enhanced dendritic arborization observed in the 0.1 and 1 mg/kg/d, but not 6 mg/kg/d, groups [117]. In vitro studies demonstrate that PCB 95 also enhances dendritic arborization in primary rat hippocampal and cortical neurons, confirming that this effect is mediated independent of systemic effects of PCBs (reviewed in [45]). The dendrite-promoting activity of PCB 95 [116,117,118,119], and another NDL congener, PCB 136 [120], have been observed in high-density neuron-glia co-cultures derived from PND 1 rat hippocampi and neocortices exposed to pM to nM concentrations, which are within the concentration range of PCB 95 detected in human brain tissue [44]. At nanomolar concentrations, PCB 95 also increases synaptogenesis in primary rat hippocampal neurons by stimulating growth and maturation of dendritic spines [121]. These morphogenic effects are selective to dendrites in that neither PCB 95 nor PCB 136 alter axonal morphology relative to vehicle controls. Similar to PCB 95-induced dendritic arborization in vivo, the dendrite-promoting activity of PCB 95 and PCB 136 exhibits a non-monotonic concentration–response relationship with increased dendritic growth triggered at nM-pM concentrations, but not at low µM concentrations [117,120]. Dendritic growth in neurons exposed to low µM concentrations resembles that of vehicle controls, and these cell cultures are viable [117,120], indicating that cytotoxicity is not the explanation for the attenuated dendrite-promoting activity observed at the low µM concentrations. The biological reason(s) for the lack of response to low µM concentrations of these PCBs has yet to be determined.

PCB 95 also promotes dendritic growth in primary mouse hippocampal and cortical neurons, although, in this species, the effect is sex dependent, with the sex specificity varying between the two neuronal cell types [51]. The differing response is thought to reflect species and regional variations in the rates of neuronal maturation [51]. Other laboratories have demonstrated that the 4-hydroxy metabolites of NDL PCBs 112, 165, and 187 promote dendritic growth in primary mouse cerebellar Purkinje cells [122]. Interestingly, this same group found that the hydroxylated metabolites of NDL PCBs 106, 121, and 159 did not enhance dendritic arborization in this same culture system; however, these PCB metabolites inhibited the thyroid hormone-induced expansion of the dendritic arbor [122,123]. Collectively, these observations suggest that not all NDL PCBs have dendrite-promoting activity. Consistent with this observation, it has been reported that PCB 66, an NDL congener with physicochemical properties very similar to those of PCB 95, has no effect on dendritic morphology in primary rat hippocampal neurons when tested at pM to µM concentrations [117].

More recently, it has been shown that PCB 11, a contemporary lower-chlorinated NDL congener, also has potent dendrite-promoting activity [49,124]. PCB 11 enhanced dendritic arborization in primary rat and mouse hippocampal and cortical neurons at concentrations as low as 1 fM (approximately 0.22 ng/mL), which is within the concentration range of PCB 11 detected in the serum of pregnant women living in northern California [49]. In addition, both the 4-OH-PCB 11 and 4-OSO_3_-PCB 11 metabolites significantly enhanced dendritic and axonal complexity in rat primary cortical and hippocampal neuron cultures [125]. Not only were PCB 11 and its hydroxylated and sulfated metabolites more potent than the legacy NDL PCBs in enhancing dendritic growth, but, unlike the legacy congeners, these contemporary PCBs also promoted axonal growth. These results suggest that PCB 11 modulates neuronal morphogenesis via different molecular mechanisms than PCB 95 and PCB 136. The findings also raise the critical question of whether developmental exposures to PCB 11 promote dendritic and axonal growth in vivo. Answering this outstanding question is vital in order to better evaluate the public health significance of these initial findings.

Mechanistic studies have shown that the dendrite-promoting activity of PCB 95 and 136 is mediated by the modulation of calcium signaling in neurons (reviewed in [45,63,73]). Early structure–activity relationship (SAR) studies revealed that NDL PCBs [126,127], but not DL PCBs [128], increase levels of intracellular Ca^2+^ and modify calcium signaling in primary neuronal cell cultures. Using pharmacologic tools to block specific Ca^2+^ channels, NDL PCBs are reported to increase neuronal levels of intracellular Ca^2+^ by activating L-type voltage-sensitive Ca^2+^ channels or NMDA receptors in the plasma membrane [129,130], and by sensitizing inositol 1,4,5-trisphosphate receptors [131] and ryanodine receptors (RyR) (reviewed in [74]) in the endoplasmic reticulum (ER). Of these mechanisms, the most sensitive is RyR sensitization. RyR channels regulate Ca^2+^ storage and release in the ER. At pM to nM concentrations, NDL PCBs bind directly to RyR channels to stabilize the channel in the open configuration [132,133]. Electrophysiological, biochemical, cellular, and in vivo techniques confirm that PCB interactions with RyR channels are governed by a stringent SAR, including stereoselectivity [120,133,134,135,136,137,138,139].

Pharmacologic and siRNA knockdown of RyR1 and RyR2 block the dendrite-promoting effects of PCB 95 and 136 in primary neuronal cell cultures [117,120]. Limited SAR studies confirm that PCB congeners with negligible RyR activity, such as PCB 66 and the (+)−enantiomer of PCB 136, also lack dendrite-promoting activity [119,120]. In vivo observations support the hypothesis that PCB engagement of RyR are linked to PCB effects on dendritic morphology. As discussed earlier, developmental exposure of rats to Aroclor 1254 enhanced basal dendritic arborization and interfered with experience-dependent dendritic plasticity [116]. Consistent with data indicating that RyR-active NDL congeners are the predominant PCBs in Aroclor 1254 [140,141], developmental exposure to Aroclor 1254 was found to significantly increase [3H]-ryanodine binding in the same brain regions in which Aroclor 1254 altered dendritic arborization [116]. Only open RyR channels can bind ryanodine; therefore, increased ryanodine binding indicates that Aroclor 1254 increased RyR activity [74]. Importantly, the dose–response relationship for Aroclor 1254 effects on RyR activity and expression and dendritic morphology overlapped [116].

**Figure 1:**
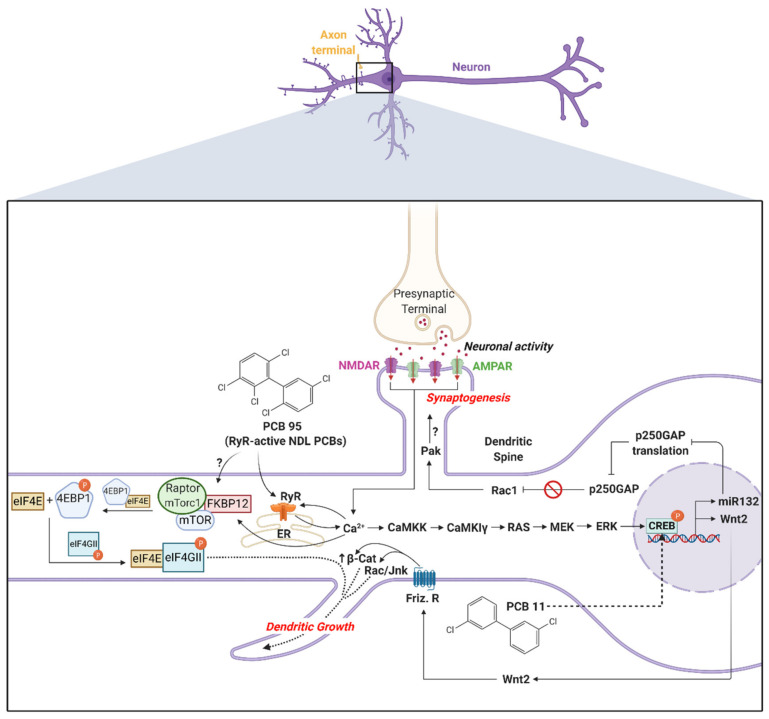
Schematic representing the signaling pathways that mediate the effects of polychlorinated biphenyls (PCBs) on dendritic morphology. Created with BioRender.com.

Calcium signaling regulates activity-dependent dendritic growth and synapse formation via both translation- and transcription-dependent mechanisms [142,143,144]. PCB 95 enhances dendritic growth by hijacking these same signaling pathways downstream of glutamate receptors (Figure 1). In primary rat hippocampal neurons, the PCB 95 sensitization of RyRs triggers the release of intracellular Ca^2+^ from the ER, which activates the mechanistic target of rapamycin (mTOR) signaling to initiate translational mechanisms that stimulate dendritic growth [118]. The increase in intracellular Ca^2+^ caused by the PCB 95 sensitization of RyR also triggers the sequential activation of calcium/calmodulin-dependent protein kinase kinase (CaMKK), calcium/calmodulin-dependent protein kinase (CaMKIα/γ), rat sarcoma virus (RAS), mitogen-activated protein kinase kinase (MEK)/extracellular signal-regulated kinase (ERK) and cAMP response element binding protein (CREB) to increase the transcription of wingless-type MMTV integration site family, member 2 (Wnt2), which acts in an autocrine fashion to enhance dendritic growth [119]. Pharmacological inhibition of RyR activity blocks the activation of these signaling molecules, and experimental manipulations to inactivate the signaling molecules block PCB 95-induced dendritic growth [118,119]. PCB 95 activation of CREB also upregulates miR132, which suppresses the translation of p250GAP to enhance dendritic spine density and increase the frequency of miniature excitatory post-synaptic currents [121] (Figure 1).

Blockade of RyR activity does not inhibit PCB 11-induced dendritic growth in primary hippocampal neurons [125], consistent with the weak RyR activity of PCB 11 [37]. The dendrite-promoting activity of PCB 11 is also not altered by the inhibition of signaling through the AhR or thyroid hormone receptor, but it is blocked by pharmacologic inhibition or siRNA knockdown of CREB [125] (Figure 1). Interestingly, even though the proximal signaling events mediating the dendritic effects of PCB 11 are divergent from the RyR-dependent mechanisms by which NDL PCBs promote dendritic growth, both PCB 11 and the RyR-active NDL PCBs converge on CREB signaling to enhance dendritic arborization. These observations suggest the intriguing possibility that genetic polymorphisms in CREB signaling present relevant targets for stratifying epidemiological studies of PCB developmental neurotoxicity.

Preclinical studies demonstrate that PCBs also interfere with synapse formation and normal patterns of neuronal connectivity in the developing brain. Developmental exposure to PCB 95 alters the topographic organization of the primary auditory cortex of weanling rats to create an imbalance between excitation and inhibition [145]. Proteomic studies reveal that NDL PCBs increase the expression of synaptic proteins in rat cerebellar neurons [146], while morphometric and electrophysiological studies demonstrate that RyR-active PCBs increase dendritic spine formation and synaptogenesis in rat hippocampal neurons [121]. In primary rat neurons, PCB 95-induced synaptogenesis coincides with upregulated miR132 expression and inhibition of miR132 activity by antisense oligonucleotides blocks the synaptogenic effects of PCB 95 [121]. While dendritic spine density is often positively correlated with cognitive capacity, histological analyses of brains from individuals diagnosed with ASD [110,147] or fragile X syndrome [148] reveal significantly increased dendritic spine densities relative to neurotypical controls. If PCBs cause similar effects in children, it seems plausible that PCBs might contribute to the deficits in communication and social interactions that are core to ASD. Furthermore, these data suggest that hyperconnectivity may be as disruptive to normal cognitive function as hypoconnectivity.

## 4. Mechanisms by Which PCBs Might Influence ASD Risk

**Figure 2:**
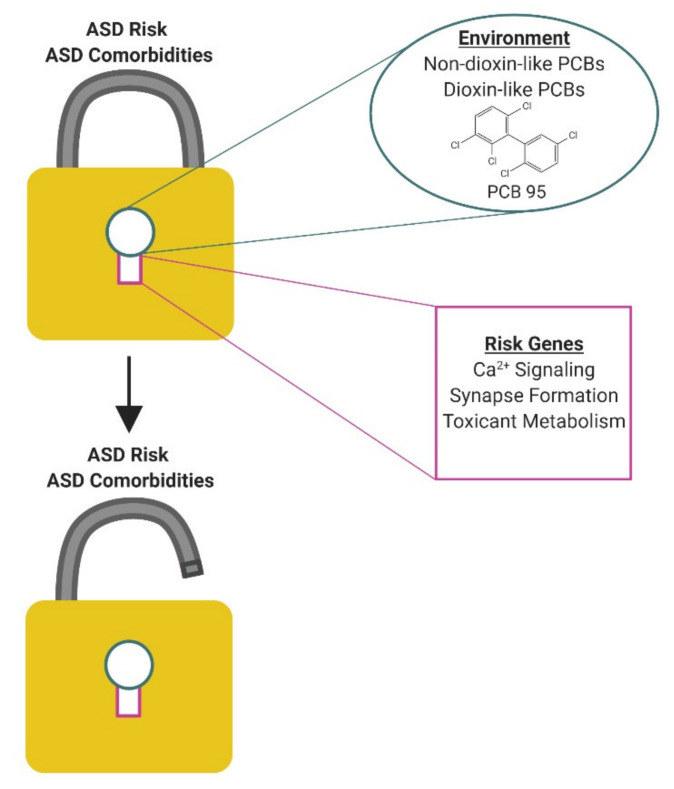
Lock and key model of ASD. The risk for autism spectrum disorder (ASD) and its comorbidities can be thought of as a lock that opens when the keys of environmental exposure to PCBs, risk genes, and timing fit into the lock key-hole. Each individual’s lock may be slightly different, contributing to the clinical heterogeneity of ASD. Created with BioRender.com.

While epidemiologic studies have linked developmental PCB exposures to an increased risk of ASD in diverse populations [51,66,67,68,149], these associations are not strong. This is consistent with a model in which PCBs interact with genetic susceptibilities to influence ASD risk rather than directly causing ASD (Figure 2). Human studies are just beginning to uncover gene x environment interactions of relevance to ASD. For example, the expression of variants in the MET receptor tyrosine kinase gene, which is important in regulating neuronal differentiation [150], were recently linked to increased ASD risk following developmental exposure to high levels of air pollution [151]. While human studies have yet to identify specific genes that interact with PCBs to influence ASD risk, the human literature does provide some hints. Post-mortem analysis of commonly detected PCBs and polybrominated diphenyl ethers (PBDEs) in brain tissue from individuals with ASD identified significant group differences only for PCB 95, which was found at higher levels in the brains of individuals with genetic forms of autism, specifically 15q11-q13 duplication (Dup15q), compared to neurotypical controls or autism of unknown etiology [44]. Subsequent in vitro studies found that PCB 95 differentially modified the methylation of genes involved in chromatin regulation and neuronal synapses in human neuronal cells expressing Dup15q [152]. These observations suggest the intriguing hypothesis that PCBs increase ASD risk in individuals expressing the Dup15q mutation, but whether the combined effects of PCB 95 and Dup15q on DNA methylation influence clinical phenotype has yet to be demonstrated.

ASD risk genes that converge on molecular and cellular mechanisms implicated in PCB developmental neurotoxicity are lead candidates for interacting with PCBs to increase ASD risk and/or influence phenotypic expression in ASD (Table 1). Of significant interest are heritable mutations that alter the fidelity of calcium signaling in neurons. Numerous ASD risk genes are associated with dysregulated calcium signaling [153,154,155,156]. For example, multiple ASD risk genes encode for voltage-gated and ligand-gated ion channels that regulate intracellular calcium levels in neurons. A particularly interesting example is the gain-of-function missense mutation in the L-type calcium channel Ca(v)1.2 that causes Timothy Syndrome. Timothy Syndrome has a 60% rate of co-morbidity with ASD [157], making it one of the most penetrant monogenic forms of ASD. Induced pluripotent stem cells (iPSC)-derived neurons from Timothy Syndrome patients exhibit increased neuritic complexity and elevated expression of genes linked to Ca^2+^-dependent regulation of CREB, including CaMK [158]. These upregulated calcium-dependent signaling molecules map onto the signaling pathway shown to link PCB sensitization of RyR to increased dendritic arborization [119]. A second example is *FMR1* premutation (55–200 CGG repeats in the 5′ non-coding portion of *FMR1*), which is the most prevalent single gene disorder associated with increased ASD risk [159,160]. Hippocampal neurons cultured from an *FMR1* premutation knockin mouse model exhibit aberrant electrical spiking patterns and synchronized Ca^2+^ oscillatory behaviors [161] coincident with impaired dendritic growth and complexity [162] that phenocopy the effects of PCBs on primary hippocampal neurons derived from postnatal rodents [144]. These observations suggest the testable hypothesis that the expression of mutations that increase the rate and/or amplitude of Ca^2+^ oscillations amplifies the neuronal response to PCBs that similarly alter Ca^2+^ fluxes, thereby increasing the likelihood and/or magnitude of functionally significant changes in calcium-dependent neurodevelopmental processes that determine the patterning of neuronal connections in the developing brain.

ASD has also been linked to the dysregulation of a number of genes whose gene products are tightly regulated by calcium signaling, including CREB, mTOR, and Wnt [166,167,168,169,170,171]. These signaling molecules are of particular interest because they function in calcium-dependent signaling pathways that have been causally linked to PCB effects on dendritic arborization [118,119,125]. Recent observations suggest that CREB is a convergent target for PCB congeners that promote dendritic growth via RyR-dependent mechanisms, such as PCB 95, as well as lower-chlorinated PCBs such as PCB 11 that also enhance dendritic arborization but have negligible RyR activity [73]. Dysregulated CREB signaling has been documented in ASD individuals [168,172], and transgenic mice expressing human mutations in CREB-binding protein exhibit increased stereotypy as well as deficits in social and cognitive behavior [173]. One downstream effector of CREB activation is miR132, which has been shown to be elevated in ASD [174,175,176] and has been implicated in PCB-induced synaptogenesis [121]. Wnt, another downstream effector of CREB, is also implicated in the pathogenesis of ASD, and is thought to underlie the stereotypic, repetitive behaviors observed in ASD [166,167]. There is also evidence suggesting that disrupting Wnt signaling in a rat model impairs social behaviors as a result of changes in brain organizational patterning and interhemispheric connectivity that are reminiscent of ASD [177]. Collectively, these observations identify CREB signaling as an intriguing target for future studies investigating gene X PCB interactions in ASD.

A second major group of ASD risk genes that may interact with developmental PCB exposures to confer increased ASD risk and/or severity are genes involved in the formation, elimination or plasticity of dendrites, axons, or synapses. Genetic, histologic, electrophysiological and functional imaging studies of children and adults with ASD all point to altered patterns of neuronal connectivity in the developing brain as the neurobiological substrate underlying these disorders [13,15,28,102,112,164]. A number of neurodevelopmental processes ranging from early events of cell proliferation and differentiation to migration and axonal outgrowth to late events involving neuronal apoptosis, refinement of the dendritic arbor and synapses, and myelination are critical determinants of neuronal connectivity in all brain regions, including those involved in ASD. Even subtle shifts in the spatiotemporal patterns or magnitude of any of these events can interfere with the formation of meaningful networks resulting in deficits in functional connectivity [104,107,178]. Data from genetic studies of ASD and neuropathological evidence from syndromic disorders with a high incidence of ASD diagnosis, such as Fragile X syndrome, Timothy syndrome, and tuberous sclerosis, suggest that the later stages of neurodevelopment, particularly dendritic growth and synaptogenesis, are probably most vulnerable in ASD [13,17]. This is consistent with the usual diagnosis of ASD within the first three years of life, during which time there is extensive formation and refinement of synaptic connections in the human brain [179]. Studies of animal models expressing ASD risk genes further support the hypothesis that the altered neuronal connectivity observed in ASD reflects perturbations of dendritic growth, synapse formation and synapse stabilization [13,100,101,102,108]. RyR-active PCBs also promote basal dendritic growth in hippocampal and cortical neurons, both cell types of relevance to ASD, in vivo [114,115,116,119] and in vitro [116,117,118,120]. These effects phenocopy the enhanced dendritic arborization and neuronal connectivity observed in at least a subset of individuals with ASD [108,112,147]. These results suggest that the convergence of both genetic risk factors and PCBs on neuronal structure and connectivity may be a way in which the genetic substrate and the environment interact to influence individual ASD risk. Another genetic factor of particular interest to gene x environment interactions in terms of connectivity is the role of miR132, which has been shown to be elevated in ASD [174,175,176]. As discussed above, PCB 95-induced increases in synaptogenesis in primary rat neurons is driven by upregulated miR132 expression [121]. In vivo studies in preclinical models indicate that while miR132 is required for cognitive function, the overexpression of miR132 to supra-physiological levels compromises cognitive function coincident with significantly increased spine formation [180]. These data suggest that gain-of-function mutations in miR132 may interact with PCBs to increase ASD risk and/or severity.

Another mechanism by which genetic risk factors may interact with PCBs to influence ASD risk and/or severity is through genetic influences on PCB metabolism. It is well established that genetic polymorphisms in paraoxonase (*PON*) influence the detoxification of neurotoxic pesticides [181], while mutations in genes encoding metal transport proteins (e.g., *SLC11A3* or *MTF1*) alter the disposition of metals in the brain and other target tissues [165]. A recent meta-analysis of two high-risk prospective cohort studies that analyzed cord blood from children with ASD versus typically developing controls found enrichment in toxic substance response and xenobiotic metabolism genes among the ASD-associated nominally differentially expressed genes [182]. Genes in this ontogeny included cytochrome P450 monooxygenases, which are involved in human metabolism of PCBs [183]. In light of experimental evidence demonstrating that hydroxylated PCB metabolites alter dendritic arborization in vitro [122,123,125] and increase reactive oxygen species and cell death in the rodent cerebellum [184,185], these data suggest the possibility that PCBs may interact with polymorphisms in cytochrome P450 to modulate individual risk for ASD. Other studies have similarly identified polymorphisms in genes related to toxicant metabolism enriched in individuals with ASD compared to neurotypical controls [165]. One of these ASD-linked genes was glutathione-S-transferase, polymorphisms of which have previously been shown to interact with PCBs to determine the risk of endometriosis [186,187]. Interestingly, a recent exploratory study observed a trend towards a positive association between PCB 153 and ASD in individuals with a deletion mutation in glutathione transferase but not in those who did not have this mutation [66]. These preliminary observations need to be confirmed in studies adequately powered to examine the impact of PCB exposures on autism risk in a population stratified by genotype for xenobiotic metabolism enzymes, such as glutathione transferase. These types of genetic studies are challenging in humans because, even when polymorphisms in genes responsible for toxicant metabolism are identified, they are not always linked to changes in function of the gene product, and, conversely, changes in enzyme activity can occur without any indications of genetic differences [165]. Future human studies aimed at linking genetic variants with enzyme activity in the context of documented PCB exposure levels will help to address these key data gaps to more directly understand whether and how genetic variants in xenobiotic metabolism and toxic substance response interact with PCB exposures to influence ASD risk.

Relevant preclinical animal models are crucial to confirming and informing human studies of gene x environment interactions in ASD. The utility of animal models in this context is elegantly illustrated in studies of mice harboring a single allele mutation in the sonic hedgehog (*Shh*) gene, which is typically silent. Relative to wild-type controls, *Shh* mutant mice were found to exhibit enhanced sensitivity to the teratogenic effects of the pesticide synergist piperonyl butoxide [188]. Similarly, point mutations in *RYR*, which are silent under normal physiologic conditions, confer susceptibility to malignant hyperthermia (MH) triggered by exposure to halogenated anesthetics [74]. It is estimated that ~35% of the human population carry one or more *RYR1* or *RYR2* variants [189]. While any one of the ~180 known human *RYR1* mutations may be rare, mechanistic studies of individual mutations indicate that they confer susceptibility to MH via a common mechanism of Ca^2+^ dysregulation [74]. These same *RYR* mutations also confer heightened sensitivity to RyR-active PCBs [190]. PCB 95 disrupts the normal homeostatic mechanisms that regulate RyR channel opening and calcium release, such that RyR channels become hyposensitive to inhibition by high concentrations of Mg^+^ and Ca^2+^ and therefore are stabilized in their open configuration [190]. In RyR1 channel preparations from pigs with either wild-type *Ryr1* or a human MH mutation in *Ryr1* (R615C), PCB 95 enhanced activity in mutant RyR1 channels relative to wild-type channels: the IC_50_ for Ca^2+^ inhibition in wild-type RyR channels increased 10-fold in the presence of PCB 95 compared to vehicle, while, in the mutant RyR channels exposed to PCB 95, it increased 20-fold compared to vehicle [190]. Together these findings indicate that MH mutations enhance the sensitivity of RyR to PCB 95, and provide proof-of-principle evidence that the genetic substrate influences functional responses to PCBs. Moreover, a joint association test of results from a genome wide association study identified *RYR2* as an ASD candidate gene by using sex as an additional risk factor [191].

Mouse models that express genes that result in dysregulated calcium signaling are of particular interest to understanding the influence of gene x PCB interactions in ASD. These include mice engineered to express human MH mutations in *Ryr1* [192,193] or the human CGG repeat expansion in the premutation range in the fragile X mental retardation (*Fmr1*) gene [156]. Relative to congenic wild-type controls, mice that express these mutations exhibit altered dendritic morphology in hippocampal and cortical neurons coincident with altered social behavior [194]. The effects on dendritic arborization phenocopy the effects observed with developmental PCB exposure alone [116]. These genetic mouse models are currently being used to test the hypothesis that the expression of these heritable mutations that alter the fidelity of Ca^2+^ signaling influence the developmental neurotoxicity of PCBs. Initial findings with these mice exposed developmentally to a PCB mixture that mimics the congener profile detected in the serum of pregnant women at increased risk of having a child with ASD [37,195] indicate that expression of both the mutant *Ryr1* and *Fmr1* CGG repeat expansion mutation enhances sensitivity to PCB effects on intestinal physiology, inflammatory markers, and the microbiome [196]. Additional findings from these translational animal models related to effects on neuronal connectivity and behavior should be informative for human studies aimed to determine whether PCBs interact with genetic mutations to increase ASD risk.

## 5. The Peripheral Nervous System: A Point of Convergence between ASD and PCBs?

Evolving evidence suggests that the peripheral nervous system (PNS), and, in particular, the autonomic nervous system, may contribute to the clinical symptoms associated with ASD [197]. There are reports of abnormal parasympathetic function in ASD during rest and while performing mental tasks [198]. Heart rate variability, which is used to measure autonomic function, is lower in children with ASD versus controls [199]. These findings were confirmed in a recent meta-analysis that also concluded individuals with ASD exhibited lower heart rate variability versus controls under conditions of social stress [200]. Autonomic pathways involved in facial recognition tasks are abnormal in children with ASD compared to neurotypical controls, with deficits including both hypo- and hyperresponsive sympathetic activity [201]. Peripheral nervous system responses measured by electrodermal activity are more rigid in individuals with autism versus controls when viewing faces accompanied by fear odors [202]. Children with ASD also display differences in sympathetic nervous system function during sensory challenges, exhibiting enhanced taste and smell sensitivity [203]. In a similar manner, mouse models that express ASD-linked mutations in *Mecp2*, *Gabrb3*, *Shank3*, and *Fmr1* have been shown to exhibit hypersensitivity to tactile stimuli [204]. Recent evidence also identifies impaired enteric nervous system function in valproic acid rat models of ASD with Vitamin A deficiency [205].

PCBs have been reported to decrease sensory and motor nerve conductance velocity in humans exposed to high levels of PCBs [206], and in vitro studies of sensory neurons isolated from chick dorsal root ganglia support a direct toxic effect of PCBs on these peripheral neurons [207]. Epidemiologic studies of populations living in highly polluted areas of northern Italy or Anniston, Alabama in southeastern United States found an association between PCBs and increased rates of hypertension [208,209]. Since hypertension is often associated with increased sympathetic tone [210], these observations suggest the possibility that PCBs increase hypertension via effects on the sympathetic nervous system. Consistent with this possibility, unpublished data suggest that PCBs enhance the dendritic arborization of primary sympathetic neurons in culture via RyR-dependent mechanisms (Panesar and Lein, personal communication). Increases in the size of the dendritic arbor of postganglionic sympathetic neurons is directly correlated with sympathetic tone [211] and has been causally linked to hypertension [212,213]. These observations suggest that additional human and preclinical studies to evaluate the effects of PCBs on the connectivity and function of autonomic and sensory nerves are warranted.

Another plausible factor linking ASD, PCBs and the PNS is the gut microbiome. Emerging evidence suggests that bidirectional communication between the gut microbiome and brain is mediated in part by the enteric nervous system [214]. While the enteric nervous system is distinct from the autonomic nervous system, it is similar to the sympathetic and parasympathetic nervous systems in that it is derived from the neural crest [215]. There is growing clinical evidence that an altered gut microbiome is associated with ASD [216,217], and recent studies demonstrate that PCBs can alter the gut microbiome [218,219,220,221,222]. For example, in one of the latter studies [222], developmental exposure of mice to a PCB mixture that mimics the PCB congener profile found in the serum of mothers at increased risk of having a child with ASD [37,195], altered intestinal physiology in weanling mice, coincident with changes in the microbiome [196]. Whether the PCB effects on intestinal physiology are linked to changes in the enteric nervous system remains to be determined, but, collectively, these observations suggest that the question of whether PCBs contribute to deficits in autonomic and sensory function in individuals with ASD is deserving of further exploration.

## 6. A Role for PCBs in ASD Comorbidities?

Developmental PCB exposures have been linked to disruptions in the microbiome and intestinal physiology [222], liver function [223,224], lipid metabolism [225], and systemic inflammation [226,227]. These outcomes overlap with comorbidities common in ASD [1], raising the question of whether developmental PCB exposures contribute to ASD comorbidities. One comorbidity that significantly impacts quality of life for children and adults with ASD, and for their caregivers, is bladder and bowel dysfunction, the incidence of which is significantly increased in ASD [228,229]. While much more is known about bowel dysfunction [230] compared to bladder dysfunction in this population, bladder dysfunction in individuals with ASD is gaining clinical interest. One study that examined both bowel and urinary incontinence found that 40% of children with ASD experience some type of urinary or bowel incontinence compared to 4.7% in typically developing controls [231]. Further examination of bladder incontinence alone identified nocturnal enuresis (bed wetting) rates of 30% and daytime urinary incontinence rates of 25% in children with ASD [231]. Urinary incontinence is not unique to ASD. It is estimated that 20–40% of children with urinary incontinence fit criteria for a psychiatric disorder [232]. In a twin study that examined rates of other health problems in children with neurodevelopmental disorders, including ASD, ADHD, or learning disability, daytime enuresis (daytime incontinence) was found to be significantly more common in individuals with a diagnosed neurodevelopmental disorder than in controls [233]. In children with a diagnosis of autism and learning disability, the prevalence of daytime enuresis was 14 times greater, reaching rates of nearly 50% [233]. Incontinence is also a common comorbidity among children with ADHD, and, in one study of ADHD and nocturnal enuresis, genetic factors alone could not account for the high comorbidity, suggesting that environmental factors play a role [234,235]. While additional large genetic studies are needed to understand the relative contributions of genetics versus environmental factors of bladder dysfunction in ASD, the role of environmental factors, such as PCBs, in determining urinary incontinence in ASD is an exciting new avenue of study.

The hypothesis that environmental factors contribute to the high rate of bladder dysfunction in ASD is strengthened by evidence that various environmental chemicals have been shown to alter urinary function in preclinical models [236,237,238]. For example, the estrogenic chemical bisphenol A has been linked to abnormal lower urinary tract function [236,239]. Several PCBs have been identified to have estrogenic activity [96], but whether they alter urinary function is unknown. Of particular relevance to the question of whether developmental PCB exposures alter bladder function, developmental exposure to TCDD has been documented to disrupt lower urinary tract function [237,238]. These effects may be modulated by the genetic substrate. In mice genetically susceptible to developing prostate neoplasia, developmental TCDD exposure decreases bladder voiding pressure once mice reach adulthood and exacerbates lower urinary tract dysfunction when adult mice are challenged with sex hormones that mimic the aging process [238]. Testosterone and estradiol treatment of adult male mice triggers lower urinary tract dysfunction with prostate enlargement. If the animal is developmentally exposed to TCDD, these effects of sex hormone treatment in adulthood are exacerbated and the clinical profile expanded to include increases in bladder volume, prostate proliferation, prostate smooth muscle thickness, prostate and bladder collagen density, and prevalence of hydronephrosis [238]. In wild-type mice, developmental exposure to TCDD followed by adult challenge with sex hormones also results in abnormal voiding physiology, evidenced as increased frequency of small urination events compared to mice with sex hormone treatment alone [237]. Developmental TCDD exposure acts in parallel, rather than synergistically, with adult sex hormone challenge to exacerbate voiding dysfunction since proteomic analysis reveals differential protein expression patterns between the two treatments [237]. A total of 102 proteins are differentially expressed in mice exposed to TCDD and sex hormones compared to mice treated with sex hormones alone. These differentially expressed proteins belong to several biological pathways of relevance to PCB developmental neurotoxicity, including dendrite development and morphogenesis, xenobiotic metabolism, regulation of synapse structural plasticity, regulation of muscle adaptation, and calcium dependent cell matrix adhesion [237]. Perhaps the most striking finding is that the protein most strongly increase in the TCDD + hormone group compared to hormone only group is RyR1 [237]. The upregulation of RyR1 raises the intriguing hypothesis that developmental TCDD exposure followed by adult exposure to sex hormones may enhance sensitivity to the neurotoxic effects of RyR-active PCBs. While these studies focused on male mice and the involvement of the prostate in urinary obstruction and abnormal voiding dynamics, whether TCDD also alters female voiding dynamics and whether chemicals that target RyR, such as the RyR-active PCBs, impact bladder or prostate biology are areas of future study. The evidence that other AhR agonists such as TCDD impact voiding function, as well as the presence of RyR in muscle of the lower urinary tract [240], suggest the possibility that DL and RyR-active PCBs act directly on the lower urinary tract to alter voiding function. More broadly, these studies support the hypothesis that environmental exposures contribute to the variable clinical profile of comorbidities in ASD, even in individuals with similar genetic profiles.

**Figure 3:**
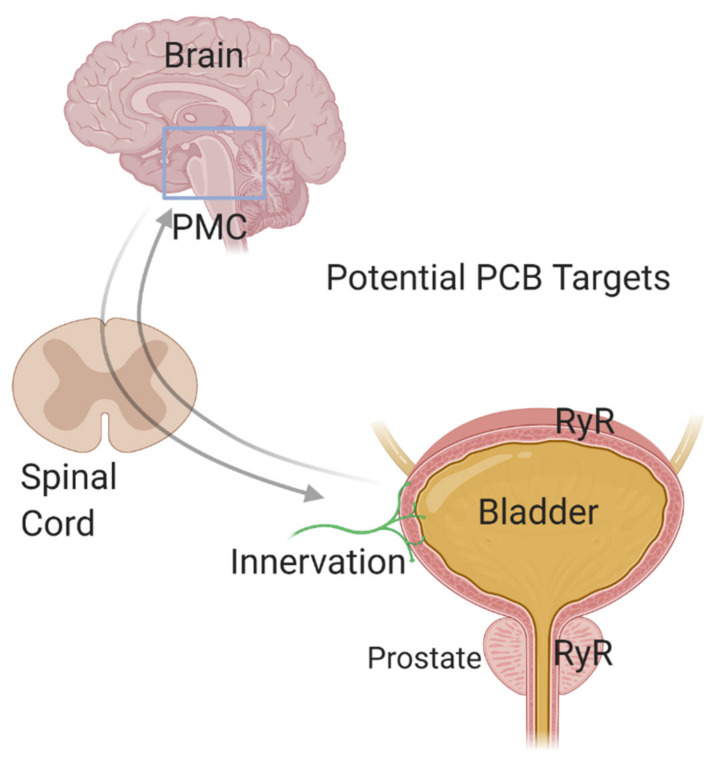
Potential PCB targets that might contribute to bladder dysfunction observed in ASD. PCBs could alter central control of micturition, sensory or autonomic control of bladder function and/or function of target organs, such as smooth muscle in the bladder and prostate, which express RyR. Created with Biorender.com.

To fully understand the pathogenesis of ASD comorbidities, greater knowledge of how PCBs and other environmental factors influence the function of peripheral targets is needed (Figure 3). Autonomic disturbances have been linked to gastrointestinal, bladder, and immune dysfunction [214,241,242], suggesting that the PNS may be a convergence point through which environmental factors such as PCBs influence the phenotypic expression of ASD comorbidities. In the context of the bladder, parasympathetic and sympathetic neurons innervate the bladder to control the micturition reflex, and sensory afferent signaling is responsible for the sensation of bladder fullness as well as responses to noxious stimuli within the bladder [243,244]. Thus, PCB-induced perturbations in the “wiring” or synaptic connectivity of the PNS during development could contribute to bladder dysfunction. The voiding reflex also has central nervous system components including Barrington’s nucleus or the pontine micturition center (PMC) [245]. Studies have demonstrated that calcium flux into neurons within this region correlate with voiding [245]. Additionally, the PMC receives input from other brain regions, including prefrontal, motor and somatosensory cortex, the hypothalamus, and the midbrain [245]. The well-established role of RyR-active PCBs in sensitizing RyR in hippocampal and cortical neurons to increase calcium release, which then alters dendritic arborization and synapse formation, raises the possibility that PCB-induced changes in connectivity within the PMC or other centers in the brain that regulate bladder function may also contribute to voiding dysfunction in individuals with ASD.

The above discussion raises a challenge and an opportunity with regards to studying the effects of PCBs on peripheral target tissues linked to ASD comorbidities, such as the bladder, in that it is conceivable that PCBs might alter bladder function via direct effects at the level of the bladder, the PNS, or the CNS, or via effects on a combination of these targets (Figure 3). This is a current challenge but with complementary approaches that employ ex vivo physiology techniques to examine the bladder alone [246,247], mouse models that allow for the direct labeling of a subset of PMC neurons important for voiding [245], voiding function physiology testing in live animals [248,249,250] and in vitro techniques to culture sympathetic and sensory neurons [251,252], we can begin to understand this complex puzzle. Such studies will provide a clearer picture of how PCBs not only contribute to ASD risk but how they may also contribute to the comorbidities associated with ASD that so greatly impact quality of life.

## 7. Conclusions and the Path Forward

Experimental studies in preclinical models demonstrate that PCBs modulate Ca^2+^-dependent signaling pathways implicated in ASD and these biochemical effects are linked to changes in dendritic arborization and synaptic connectivity that phenocopy morphometric changes observed in autistic patients and in animal models that express ASD risk genes. However, a critical observation emerging from mechanistic studies of PCB developmental neurotoxicity is that not all PCB congeners exhibit these activities. Not only are there differences between congeners, but also between parent compounds and their metabolites. The question of whether and how PCB metabolism influences PCB developmental neurotoxicity remains an outstanding question in the field. Given the known differences in human vs. rodent metabolism of PCBs [87,253,254,255,256,257], it will be important to address species differences in translating data from rodent models to human risk. One potential approach for overcoming this challenge would be to use “humanized” mice [258,259,260,261,262,263] that not only express relevant human cytochrome p450 isoforms [87,264,265,266] but also lack expression of rodent cytochrome P450 enzymes involved in PCB metabolism. Another critical gap is whether the in vitro effects of PCB 11 on neuronal morphogenesis [49,125] translate to the intact developing brain and whether they can be generalized to other contemporary lower-chlorinated PCBs found at relatively high abundance in the serum of pregnant women, such as PCB 28 [37].

Since PCBs likely do not cause ASD per se but rather modify genetic risks of ASD, this observation may explain in large part why epidemiologic studies have found only weak associations between PCBs and increased autistic traits. Key strategic goals moving forward are (1) to test a greater number of individual PCB congeners, particularly those currently identified to be most prevalent in relevant human tissues, (2) determine the SAR for PCB effects on neuronal connectivity, and (3) confirm the molecular mechanisms mediating the effects of PCBs on neuronal connectivity and determine whether and how these map onto ASD risk genes. Such information will be of immense value in optimizing PCB analyses conducted in epidemiologic studies, helping to shift from the current strategy towards testing associations within structural and mechanistic classes of PCBs or within specific genotypes. This information will also inform strategies to stratify epidemiology studies to more effectively identify gene x PCB interactions that increase ASD risk.

**Figure 4:**
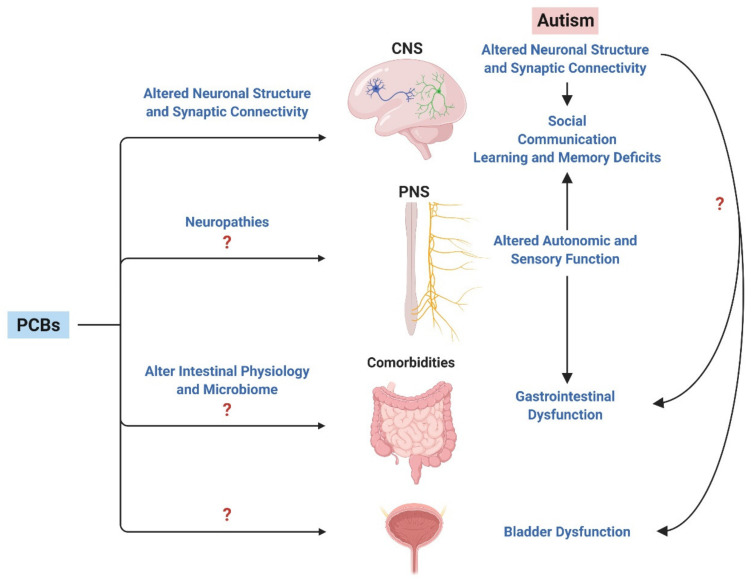
Links between PCBs and ASD. Developmental PCB exposure has been linked to altered neuronal connectivity in the central nervous system (CNS), an endpoint that is also disrupted in ASD. Growing evidence supports the involvement of the peripheral nervous system (PNS) in the behavioral deficits observed in ASD as well as comorbidities such as gastrointestinal (GI) dysfunction. The direct effects of PCBs on the PNS, GI tract and other organs, such as the bladder, are understudied, but elucidating the impact of PCBs on peripheral targets may help to explain the clinical heterogeneity that is a hallmark of ASD. Created with BioRender.com.

An emerging area of PCB research that has the potential to significantly shift the current paradigm regarding the role of environmental risk factors in ASD is the influence of PCBs on not only core symptoms of ASD, but also comorbidities commonly associated with ASD via effects on the central and peripheral nervous systems, and/or peripheral target tissues (Figure 4). This is a nascent field of inquiry, so significant data gaps need to be addressed to validate this model. However, by adapting the biological framework established for PCB effects on the CNS, it should be relatively straightforward to develop testable hypotheses regarding PCB influence on the morphometric determinants of neuronal connectivity in the PNS. Data supporting a role for PCBs in influencing the phenotypic expression of comorbidities would suggest interesting and potentially extremely important associations to test in epidemiologic studies. Increased understanding of the impact of PCBs on autonomic and sensory neurons and how these effects contribute to the risk of ASD comorbidities may provide insights into novel therapeutic approaches to greatly improve quality of life for individuals with ASD.

Clearly, work is urgently needed to better predict the specific ASD genes and specific PCB congeners that together pose the greatest ASD risk. Ultimately, data indicating that developmental PCB exposure increases the risk and/or severity of ASD core symptoms and/or comorbidities would justify the resources needed for mitigating exposure of genetically susceptible individuals. The fact that chemical exposures are more readily controlled than genetic factors to prevent or mitigate the expression of phenotypic traits related to ASD, coupled with the significant toll of ASD on individuals, their families and society, provides compelling reasons to engage in this endeavor.

## Figures and Tables

**Table 1:** Potential ASD risk genes that may interact with PCBs to increase ASD risk.

Genetic Factor	ASD-Relevant Genetic Risk Factors	Further Reading
Genetic variants or changes in expression of genes regulating calcium signaling or gene products regulated by calcium	Voltage gated calcium channels (CACNA family) Voltage gated sodium channels (SCN family) Ligand gated chloride channel (GABRB3) CGG-repeat expansions in the *FMR1* gene Calcium-dependent signaling molecules: CREB, mTOR, Wnt, CaMKI	Pasca et al., 2011 [158] Stamou et al., 2013 [28] Nguyen et al., 2018 [155] Marcantoni et al., 2020 [154] Hagerman and Hagerman, 2013 [163]
Genetic variants or changes in expression of genes regulating dendritic arborization, axonal growth, or synapses	Neuroligins (NLG3, NLG4) Neurexins (NRXN1, NRXN3, CNTNAP2) SH3 and multiple ankyrin repeat domains 3 (SHANK3) MET receptor tyrosine kinase miR132	Qiu et al., 2012 [15] Zoghbi et al., 2012 [102] Stamou et al., 2013 [28] Eagleson et al., 2017 [164] Guang et al., 2018 [13]
Genetic polymorphisms that alter metabolism or response to PCBs	Paraoxonase (PON) Glutathione S-transferases (GST) Delta-aminolevulinic acid dehydratase enzyme (ALAD2) Cytochrome P450 monooxygenases	Rossignol et al., 2014 [165] Bach et al., 2020 [66]
